# The Ambivalent Students’ Cognition to Be English Teachers for Young Learners: A Longitudinal Study

**DOI:** 10.3389/fpsyg.2022.818883

**Published:** 2022-03-17

**Authors:** Yuli Astutik, Slamet Setiawan, Syafi’ul Anam

**Affiliations:** English Department, Universitas Negeri Surabaya, Surabaya, Indonesia

**Keywords:** pre-service teachers, cognition, teaching English for young learners course, EFL context, higher education

## Abstract

This longitudinal study analyzed university students’ cognition in learning an English for young learners (EYL) course. A qualitative method was used to get the data from 28 students who took the tiered EYL courses, EYL 1, EYL 2, and EYL 3, at a private university by giving them open-ended questionnaires for three semesters, or one and a half years. Semi-structured interviews with those 28 students were also used as the triangulation data at the end of each semester. The findings indicate a very extreme change in pre-service teachers’ cognition, such as motivation, perception, and belief. At the end of their lecture, students initially interested in learning English for young learners did not want to become EYL teachers. On the other hand, students who enrolled in the EYL course for non-academic reasons wanted to have a profession as an EYL teacher after completing the EYL courses. It proves that students’ interest in teaching English to young learners and the length of time spent studying EYL teaching knowledge do not assure those pre-service teachers are increasingly convinced to have a teaching profession.

## Introduction

As a foreign language, Learning English from an early age is better than learning English as an adult because children can get more extended benefits from learning a foreign language for their future (Uysal and Yavuz, 2015; Nguyen, 2016; Nikolov and Timpe-Laughlin, 2021). Some experts assert that language is not a science. However, it is an ability that can be honed as early as possible. Many parents think that by starting their children’s education to learn English from an early age, they will have a better future. They believe that introducing English to their children as soon as possible provides them with a better educational future (Djiwandono, 2005; Dong et al., 2020; Rafiqa et al., 2021; Susanty et al., 2021). These issues encourage parents to enroll their children in the best English language schools and hire private English teachers (Fletcher et al., 2009; Coffey and Farinde-Wu, 2016; Manan and Khadija-Tul-Kubra, 2017; Leo, 2021). Learning English can be one of the exciting activities for children, making them more confident in facing their real-life and ready to face the next level of education (Tumasang, 2021).

Numerous non-formal English training institutions in Indonesia are highly professional in teaching English as a foreign language to young learners. Regrettably, informal education at the kindergarten and elementary school levels devolves into lessons on local content. Even though English is not a compulsory subject in primary school, an English subject is needed to provide primary school students to receive English lessons in secondary school later (Anam and Stracke, 2016). Therefore, many primary schools in Indonesia need qualified English teachers. Although it is not promising to a career as an English teacher in primary school, some pre-service EYL teachers are interested in working in this field.

Moreover, university students’ interest in learning pedagogy in teaching English to young learners has been steadily growing. According to the association of English language education in Indonesia, the primary profile of English language education is as an English teacher in secondary schools. On the other hand, English language education at the university level can include graduates’ profiles as EYL teachers. Thus, the English study program should include preparatory courses, precisely the EYL course, for pre-service English teachers working with young learners. As a result, many students enrolled in one of the private universities in Sidoarjo, East Java, Indonesia, choose the EYL course as their final year concentration. This course discusses student and teacher characteristics, methods for teaching English to children, lesson planning, creating teaching media for children, and practicing teaching English to children (Anif et al., 2020).

Pre-service teaching is a supervised and guided period of teaching by lecturers in education, and pre-service teachers are gradually introduced to teaching roles for specific classes. After studying teaching theory and pedagogy, they should gain classroom experience during their bachelor’s degree in college to become professional teachers (Wiggan et al., 2020). As pre-service EYL teachers, they should therefore understand a variety of concepts, including the characteristics of students who are learning a foreign language, the importance of students’ age in determining classroom teaching methods, and the theories and principles underlying the teaching of EYL (Juhana, 2014; Darwis and Hasanah, 2020). These pre-service teachers’ motivations for enrolling in EYL courses, both academic and non-academic, are undoubtedly diverse. Their varied motivations occasionally affect their cognition, like beliefs, perceptions, and knowledge during the EYL learning process, affecting their belief and desire in the EYL teaching profession. While motivation strongly influences students’ cognition, it can also increase or decrease students’ likelihood of making a decision (Finn, 2020; Kim et al., 2020). The differences in motivation have demonstrated students’ beliefs and desires about self-efficacy, preferred learning method, goals, and values in the learning process. Their response to academic tasks relating to their assignment selection, participation, and perseverance will affect their cognition (Garcia and Pintrich, 1994). This issue aligns with Levine et al. (1993, pp. 587) that the relationship between cognition and motivation has particular relevance where cognition is a source of motivation. In their work, Levine dan Resnick explained that this issue was a significant concern under discussion in the late 1950s and 1960s of social psychology, as evidenced by various models of cognitive consistency and information transmission approaches to attitude formation and modification. In addition, the work of researchers on social development has shown how different self-attributions can motivate different forms of cognitive behavior, showing how motivation can affect the form and substance of cognition.

According to Borg (2003), cognition has several dimensions: beliefs, theories, knowledge, attitudes, assumptions, conceptions, and perspectives. The term cognition refers to the non-observable cognitive aspect of teachers, which influences in making decisions for their teaching, such as what teachers know, believe, and think. Therefore, understanding the dimensions of cognition is critical for EFL teacher education, particularly for pre-service teachers preparing for a career as a teacher (Debreli, 2012; Yuan and Lee, 2014; Borg, 2015; Misrohmawati, 2016; Shooshtari et al., 2017). Even university students’ cognition, such as beliefs and perceptions, is influenced by the process of studying EYL theories and concepts, design learning instruments, and even practicum in teaching English to younger learners, which ultimately affects their desire and decision-making to become EYL teachers. Thus, a longitudinal study was conducted to track changes in cognition of pre-service teachers, particularly for teaching English to young learners in Indonesia who are currently attending English teacher education and training for young learners.

## Literature Review

### Cognition and Motivations

Cognition is an imperceptible factor influencing teachers’ instructional decisions (Borg, 2003). In contrast to Borg, who defines cognition as its influence on teacher activities when teaching, Levine et al. (1993) described cognition as a person’s essential social activity. The term cognition is derived from the phrase cognitive psychology, which was devised initially for non-social things and later tested and generalized to include social objects. Numerous cognitive models developed by social psychologists are then applied to general cognition. These include models of salience effects, accessibility of knowledge, attribution processes, inference, and decision-making. The link between cognition and motivation is relevant in the search for the foundations of social cognition (Higgins and Sorrentino, 1990 in Levine et al., 1993, pp. 587). According to Levine et al. (1993), motivation is a critical issue in social psychology, as evidenced by numerous cognitive consistency models and the information transmission approach to attitude creation and change.

Additionally, Levine et al. (1993) demonstrated how various self-attributes could inspire various types of cognitive behavior, indicating how motivation can alter cognitions such as beliefs, perceptions, desires, attitudes, and knowledge. Madan (2017) conveyed the relationship between motivation and cognition, stating that motivation impacts cognitive processes. Madan also deliberated the motivation’s influence on cognition, drawing on a variety of prior studies, such as the general-domain viewpoint on the influence of motivational elements on cognition. Madan’s findings align with Stolk and Harari’s (2014) research, which asserted that student motivation could be used to predict active cognitive involvement. The process can alter students’ knowledge, perceptions, and beliefs about a subject. In addition, in his research findings, Alieto (2019) discovered that cognition is a factor influencing the willingness of the respondents to teach in MT and to teach MT as a subject. It emphasized that cognition is a predictor of understanding students’ learning desires. Cognition is a factor that affects the willingness of students who are undergoing education as pre-service teachers to teach in specific fields.

### Cognition of Teachers and Pre-service Teachers

Numerous researchers have investigated how the cognition of teachers and pre-service teachers can change due to motivational factors, specific experiences, and even levels of understanding of the particular knowledge being studied. Concerning those changes in teacher cognition, the research has thus far focused on teacher decision-making in their classroom practice. According to Borg (2003), teacher cognition, such as beliefs, perceptions, knowledge, and feelings, are influenced by factors such as schooling, professional training, and even personal experiences during their time as teachers. Likewise, Kagan (1992) defined teacher belief is a particularly perplexing type of personal knowledge that is broadly characterized as pre- or inservice teachers’ implicit assumptions about classrooms, learning, students, and the subject matter to be taught (pp. 65–66). Samfira (2017), which focused on the dimensions of teacher beliefs, found that teachers’ practice in teaching subject matter in the classroom is influenced by their previous knowledge while taking teacher education. Samfira, on the other hand, proposed a model in which teachers’ beliefs change over time through the use of several models, including dissonance theory, conceptual change, dual processes, cognitive restructuring of knowledge, the relationship between attitude and behavior, cognitive-affective, teacher change, and the improved conceptual model. Another study conducted by Teng (2016) on the change in belief in language teachers after completing professional development training on teaching writing in Chinese. Teng found that a professional development project for teaching writing expanded teachers’ knowledge of different writing theories, which shifted their perception of the teacher’s role in their teaching practice. Those prior researches are strengthened by Richards et al.’s (2001) findings on Exploring Teachers’ Beliefs and Change Processes. Richards et al. (2001) discovered that teacher beliefs are critical to the teacher development process; changes in teaching practice result from changes in teacher beliefs; and the concept of teacher change is multifaceted, influenced by both personal and professional aspects. Thus, it is abundantly evident that changes in teacher cognition affect the teacher’s work on the practice of instruction. It means that teachers’ experiences affect their cognition, and their cognitions affect the activities of teachers in the classroom.

In contrast to changes in teacher cognition which tend to be about the impact on making decisions in their teaching practice, changes in cognition to pre-service teachers tend to discover their attitudes, beliefs, and perceptions, which can be for the better or vice versa, which makes them doubt about specific knowledge that is being studied. The longitudinal research conducted by Hollingsworth (1989) on changes in pre-service teachers’ cognition about reading instruction before, during, and after educational programs discussed dimensions of belief and knowledge to determine changes in pre-service teachers’ beliefs about education, teaching, and learning and specific knowledge about managing, assessing, and facilitating learning through texts. He used a total of 14 pre-service teachers as research participants, starting with the pre-service teachers who entered the pre-service teacher education program, attending reading classes on their campus, and then practicing teaching reading skills to high school students. The results of Hollingsworth’s research showed that the beliefs of pre-service students experience changes in the pre-program class that affect their knowledge of the main concepts of participatory and constructive learning. As a result, pre-service teachers show a less broad understanding of student learning concepts.

Another study on changes in cognition of pre-service teachers was also conducted by Yüksel and Başaran (2019), where they investigated changes in cognition of pre-service ELT teachers during teaching practicum. Yüksel and Başaran (2019) revealed the beliefs of pre-service teachers about language teaching, the dynamics of teaching practicum in the classroom, and the belief in being a teacher. The results of their research implied the complexity of the practicum process in pre-service teacher cognition. They found that some pre-service teachers changed their beliefs toward a more positive direction in their teaching practices because the practicum results provided pre-service teachers’ experience of real-life contexts. They could adapt their fundamental beliefs, acquired as language learners, and turn them into the beliefs of the language teacher. However, some pre-service teachers’ beliefs were stable and did not change during and after the practicum. The participants maintained several beliefs that worked for their better teaching practice in the future. Qiu et al. (2021) reported pre-service teachers’ belief changes before and after the teaching practice was carried out for 3 months. The results of their research showed that pre-service teachers’ beliefs were growing and had a positive impact on their future careers. Thus, the change in belief for the better could help teachers adjust appropriate education policies to improve the quality of English teacher education, especially in the Chinese context. Similar research was conducted in the Indonesian setting; Heryatun and Damanik (2018) discovered that prior learning experiences significantly contributed to pre-service teacher beliefs following the completion of the teaching practicum in the Indonesian setting. Their results indicated that teaching practice has the most significant influence on pre-service teachers’ beliefs. Moreover, this study’s findings indicated that interactions with peers and mentors influence pre-service English teachers’ beliefs.

Many studies on changes in cognition of teacher and pre-service teachers discuss ELT education in the context of high schools and colleges. However, few studies discuss teachers or pre-service teachers teaching English to young learners. In comparison, the quality of English teachers for young students must also be seen from their cognition changes as an invisible factor that needs to be known. The issues of cognition change are also experienced by pre-service teachers preparing themselves as English teachers for young learners. In carrying out education as prospective teachers, they must do teaching practicums for young learners. Even though they are only faced with children, not all pre-service teachers can maintain their beliefs about their knowledge and practice during practicum. Some of them felt nervous and realized that the theory of pedagogy in teaching English to young learners was more manageable than putting it into practice. However, some pre-service teachers have become increasingly convinced from their practicum that they can teach English to young learners better. Teaching English to young learners requires teachers who have strong beliefs in terms of knowledge and experience during teacher education. By knowing the cognition of teachers and pre-service teachers for EYL, it is hoped that education can provide good teaching for young learners. The following section discusses the importance of teaching English to young learners.

### Teaching English to Young Learners

Learning a second language or a foreign language such as English should be done young. This theory is not a new issue in the field of English Language Teaching. Numerous experts and research have revealed that children’s age is a golden age where their memory is still strong, they are easy to receive information, and they have much time to learn new things (Moon, 2005; Schröter and Danielsson, 2016; Astutik et al., 2019; Xolmurodova, 2021). This issue is undoubtedly different from people who have grown up learning English as a foreign language or second language. They generally find learning difficult due to various factors, such as not having free time to study because adult activities are more complex than children. Although children’s age is the right time to learn a language, their success in mastering English is also influenced by the quality of the teacher (Zein, 2015).

Learning English in principle is a language acquisition process that is carried out intentionally and with a purpose within the scope of the learning process in the classroom or outside the classroom by following the rules of learning. Learning English for young learners is a dynamic field, so it will inevitably experience changes. However, the task of educators for young learners is the same, namely, to help the development, understanding, and use of their language through planning, mentoring, and providing adequate and adequate support facilities. Therefore, it is essential to pay attention to the profile of an English teacher for young learners from the start. In the Indonesian setting, an English teacher for young learners needs to have five characteristics, including; (1) pre-service teachers need to have sufficient English skills; (2) pre-service teachers need to have teaching skills such as conducting assessments and classroom management; (3) pre-service teachers need to have good self-control qualities such as being patient, kind, humorous, creative, and high-spirited; (4) pre-service teachers need to have professionalism toward their work as English teachers for young learners; and (5) pre-service teachers need to have an open nature to ask questions, learn, improve themselves, and try new things suitable for their students’ needs (Suyanto, 2007).

Meanwhile, in terms of competence and use of English, Suyanto (2007) agrees that English teachers for young learners must have adequate knowledge and skills in English. It is essential because the use of the mother tongue or first language is different from English. For this reason, pre-service teachers must adequately understand things such as (1) grammar, (2) English vocabulary that is appropriate to the needs of young learners, (3) correct pronunciation or speech, (4) correct intonation and stress, (5) spelling, and (6) the culture of English also needs to be included in the learning. For this reason, teachers, especially if they are going to start giving English lessons to young learners for the first time, they must understand the basics of children’s self-development and how it relates to learning English. Therefore, before actually teaching, teachers must attend special education to teach English in universities to be academically eligible to teach. They need to prepare themselves to become competent and professional teachers in this field. Unfortunately, in the self-preparation process, there are many factors experienced by prospective teachers that affect their beliefs and perceptions of teaching English to young learners.

Discussing teaching English for young learners, some researchers deliberated the EYL pre-service teachers’ beliefs in techniques, strategies, and the extent to which the pre-service teachers implemented the existing lesson plan in teaching English to children. Othman and Kiely (2016) argue that pre-service teachers’ classroom practices do not often reflect their young learners’ language learning beliefs. Hsu (2019) conducted a longitudinal study investigating the motivation changes among young rural EFL learners in Taiwan for 3 years showed a change in student motivation every year. It presented that in the first year of research, students’ motivation was very high in the following years, students’ motivation tended to decrease to the lowest. Özmen (2012), in his 4-year longitudinal study, revealed how students’ beliefs change in English education programs about learning and teaching during their time as students. His research result showed that the students’ phase of taking lessons each year brought various changes to students’ beliefs.

Those previous researchers emphasized that the teacher or lecturer’s role in subjects or courses related to student motivation is crucial in developing students’ cognition, like belief and perception in the learning outcomes. The students’ cognition is not always in line with the initial motivation before. It is slightly different from previous studies that have never discussed pre-service teachers’ beliefs, especially in English for young learners. The researchers focus on digging deeper into pre-service EYL teachers in this study. The researchers endeavor to explore students’ beliefs who took the EYL elective courses in one of the private universities in Sidoarjo, Indonesia, using a survey-longitudinal study. These university students are called pre-service teachers because they are still in their final semester, and they must do teaching practice in primary schools.

Based on these problems, this study investigates the changes in cognition of pre-service teachers in teaching English to young learners through learning EYL courses. Therefore, the research questions consider the following three parts:

1. What is the student’s motivation in taking EYL courses?
2. How do students perceive different EYL courses?
3. What are the students’ beliefs about the EYL teaching profession?

## Materials and Methods

This research explores EFL pre-service teachers’ motivation and cognition during EYL courses using longitudinal, especially a panel study. Longitudinal analysis is social research that compares changes in research subjects after a certain period (Laurie and Lynn, 2009). This type of research is consciously used for long-term research. The researchers used the same respondents in different periods, so this research is called a panel study (Lee et al., 2019). This study followed several investigations to obtain data consistent with the same respondents (Peacock, 2001).

### Research Participant

In this research, the data sources were 28 students who took EYL courses in the English department at Universitas Muhammadiyah Sidoarjo. They consist of twenty-three females and five males with an average age of 21–22.

### Data Collection

Researchers gathered the data three times in different semester periods using an open-ended questionnaire distributed to respondents at the end of each semester in the EYL course. This EYL course is divided into three consecutive semesters in the third and fourth academic years (2018/2019–2019/2020). The names of the EYL courses and materials given at each level were: EYL 1, which focuses on the basics of EYL theory and concepts, student and teacher characteristics, methods, techniques, and EYL teaching strategies for EFL. Then, the EYL 2 course focuses on learning devices such as compiling lesson plans and EYL learning media. Before the final examination, students practiced teaching with peers at the end of the meeting, and others became observers (peer observation). The last was the EYL 3 course, where students directly teach English to primary school pupils. In doing so, researchers need one and a half years or three consecutive semesters, such as the EYL 1 course in the 6th semester of the 2018/2019 academic year, the EYL 2 class in the 7th semester of the 2019/2020 academic year, and the EYL 3 course in the 8th semester of the 2019/2020 academic year.

### Data Analysis

The questionnaires’ data were analyzed in several stages: First, reading the respondent answer from surveys at EYL 1, EYL 2, and EYL 3, then extract the answers into the text. Then, coding and classifying respondents’ answers into phrases. Third, categorizing the coding data; at this stage, the researcher then classifies the data to reduce the number of different pieces in the analysis based on the research objective. After categorizing data was done, researchers determined some themes. Themes are high-level categorization primarily to identify the main elements of the analysis that have been carried out in the previous stages. Those themes are students’ motivation to take the EYL course, perceptions on different EYL course levels, and students’ belief in the EYL teacher profession. Finally, reporting and making conclusion. The researchers adapted the coding process from Huhtala et al. (2019) and followed Saldaña (2013).

After coding and categorizing, the researchers determined three themes (Table 1): The first theme is motivation in taking the EYL course. The researchers described students’ reasons when choosing EYL courses. Second, students’ perceptions on different EYL course levels. The researchers investigated student perceptions about the materials given in EYL 1, EYL 2, and EYL 3 courses. Third, students’ belief in the EYL teacher profession. The researchers tried to analyze changes in students’ attitudes, thoughts, and opinions about the profession as an EYL teacher.

**TABLE 1:** Example of coding in data analysis.

	Text extract	Coding	Categorizing	Theme
Q:	What is your reason for selecting the EYL course?			
S1:	I love teaching, and I love children. I have been teaching privately for several months…	Teaching Interest	Personal academic motivation in EYL Course	Motivation in taking EYL course
S2:	Because I want to be an English teacher for Young learners later…	Avoiding other courses hg5	Personal non-academic motivation in EYL course	
S3	Actually, I wanted to take a translation course, but because many friends choose EYL so follow them			
Q:	How was your experience practicing teaching elementary students in the classroom?			
S1:	I really felt pressured during teaching practice, it turned out that it was not as easy as the theory…	Doubtful to be an EYL teacher	Personal challenges and experiences in learning EYL theory and concept	Students belief in EYL teacher Profession
S2:	I literally enjoy in practicing my skill to teach English to children….	Blissful to be an EYL teacher	Personal challenges and experience in teaching EYL to primary school pupils	

Instead of processing and analyzing the questionnaire data, the researchers conducted interviews to 28 respondents. Semi-structured interviews were used in this study because they involved a series of open-ended questions based on the topic interest that the researchers wanted to cover. The researchers asked the students a series of questions based on the data from the open-ended questionnaires. It was obligatory to clarify students’ motivations prior to and during their one-and-a-half years of participation in EYL courses’ learning process. This interview was conducted following the completion of each semester’s courses by all students.

In analyzing semi-structured interview data, the researchers carried out several stages, including; transcribing the recordings with the help of Google docs voice typing tools. Then the researchers reread and interpret the transcripts as a whole. The next step was identifying the theme (matching the answers with a predetermined topic), known as the coding stage. The next researchers did a memo to jot down new and unexpected information. The researchers then repeated those steps to ensure the validity of the data. Finally, the researchers wrote conclusion and made a report.

Researchers did the semi-interview as data triangulation to check the truth of data or information obtained by researchers from different perspectives by reducing as much as possible the bias that occurs during data collection and analysis.

## Results

After conducting a series of analyses on the data collected, the researchers discovered that some students consistently desired to become EYL teachers after graduating from EYL 1 to EYL 3. However, after completing the EYL 3 course, some students changed their minds about being EYL teachers. Naturally, these students have reasons for their move-in belief due to the thought process influenced by someone, experience, or knowledge during lectures. The following section contains a detailed discussion of the study’s findings.

### Students’ Motivation in Selecting English for Young Learners Course

Students enroll in the EYL course for various reasons, both academic and non-academic. Table 2 identifies students who chose EYL courses as their concentration study for academic and non-academic reasons.

**TABLE 2:** Motivation in taking EYL course.

Categories	Sub categories	Number of students for subcategories	Total number of students
Personal academic motivation	Interested in English Language Teaching	11	22
	Had experience in Teaching EYL	5	
	Wanted to gain a wealth of knowledge regarding the theory and practice of teaching English to young EFL students	6	
Personal non-academic motivation	Only interested in children, not in teaching English to children	4	6
	Opted out of other elective courses	1	
	Avoiding other lecturers	1	
Total			28

Table 2 describes respondents’ motivations for enrolling in EYL 1 courses. While Twenty-two students took EYL 1 for academic reasons, six students took EYL 1 for non-academic reasons.

Twenty-two students have academic reasons with details; Eleven students expressed a strong interest in English language teaching during the interviews. Then, five students stated that they had prior experience teaching English to young EFL students and desired to expand their knowledge of EYL pedagogy. Additionally, six students claimed they want to gain a wealth of knowledge regarding the theory and practice of teaching English to young EFL students. This finding is consistent with other research indicating that prior experience in a particular field can significantly influence students’ motivation (Peacock, 2001; Blossfeld et al., 2015; Lee et al., 2019). Thus, a teacher who teaches effectively can inspire his or her students to pursue careers as teachers.

Unlike the majority of students who enroll in EYL 1 for academic reasons, six students enrolled in the course for non-academic reasons. Four students stated that they are only interested in children, not in teaching English to children. While one student stated that she opted out of other elective courses, another stated that he chose EYL to avoid other lecturers. Their statement demonstrated that they chose EYL not out of interest. Usher and Kober (2012) argue that a student with a non-academic motivation in a course will find it more challenging to adhere to the learning objectives. As a result, the teacher’s method and strategy have to motivate students to focus solely on learning. Thus, even if their motivation is not academic, they can complete the course. Although only a tiny percentage of students have a non-academic motivation, teacher education should pay attention to them, particularly those who have a non-academic motivation, to become more interested in this course.

After examining university students’ motivations for enrolling in EYL courses, the researchers examined their perspectives following their participation in tiered EYL courses, as discussed below.

### Students’ Perceptions of Different Levels of English for Young Learners Course

This session discusses three distinct perspectives from students who have taken EYL 1, EYL 2, and EYL 3 courses.

Students’ responses to the EYL 1 course, which focuses on theories and concepts relating to the teaching of English pedagogical knowledge to EFL young learners, are summarized in Table 3. Twenty-eight students considered various responses; ten students stated that they gained knowledge of functional theory and concepts while teaching English to children in the EYL course. Additionally, they stated in the interview that they were enthusiastic about learning the methods and fundamental concepts of English teaching. The theories and concepts they learned in EYL 1 served as a springboard for entering the field. Likewise, they stated that the EYL 1 course materials prepared students to interact with a variety of student personalities in the English classroom.

**TABLE 3:** Personal challenges and experiences in learning EYL theory and concept (EYL 1 Course).

Aspects	Number of students
Feasible	10
Apprehensible	12
Well conveyed by the lecturer	6
Total	28

Then, twelve students stated that the EYL 1 course material was easy to understand. It is expected to help them when they became English teachers for children in the future as their profession. The interviews stated many techniques, strategies, and methods that are not boring and easy to practice in teaching English to young learners. Besides good and coherent theory and concepts in the EYL 1 course, six students argued that the EYL 1 course material was well presented and engaging. It made the students even more curious about the EYL teaching field. After being confirmed through interviews, they stated that they were very interested and made them even more curious. They also admitted that it was not easy to make learning plans and apply them later.

Table 4 sums up student feedback following completion of EYL 2 courses in which the lecturer focused on developing lesson plans and EYL learning media. Following that, students practice teaching EYL with their peers (peer teaching) using previously compiled lesson plans and teaching media, with others serving as observers (peer observation). Various student responses indicate that not all students enjoy and succeed at peer teaching; some are nervous and under pressure to actively deliver material in class.

**TABLE 4:** Personal challenges and experiences in designing and practicing lesson plan, media, and peer teaching (EYL 2 Course).

Aspects	Number of students
Fun learning	21
Less fun learning	4
Unpalatable learning	3
Total	28

Twenty-one students, out of a total of twenty-eight respondents, stated that the EYL 2 material was enjoyable and useful for those focusing on the EYL teaching field. Additionally, they stated that they were capable of effectively utilizing lesson plans and media when conducting peer teaching. While four students stated that the EYL 2 material was enjoyable, they stated that compiling and designing the teaching media was difficult. They were also unsure whether they could successfully implement the learning plans. Even three additional students expressed dissatisfaction with the material in the EYL 2 course. They struggled with developing lesson plans and media. When required to conduct peer teaching sessions with their classmates, they felt under pressure.

Students’ perceptions of the EYL 3 course are summarized in Table 5. Therefore, students should practice EYL teaching with primary school pupils while completing the EYL 3 course. Whereas most students responded positively, some saw it as a burden. Twenty-three students stated that EYL 3 taught them how to have fun and served as a valuable experience for them to pursue careers as teachers in the future. Then four students stated that EYL 3 was less enjoyable than the previous two because it constantly made them anxious and nervous. One student stated that EYL 3 is not enjoyable because teaching EYL to primary school pupils is inherently tricky, and he had recently realized that teaching children were not his field.

**TABLE 5:** Personal challenges and first-hand experience teaching EYL to primary school students (EYL 3 Course).

Aspects	Number of students
Fun teaching practice	23
Less fun teaching practice	4
Unpalatable teaching practicum	1
Total	28

Students’ perceptions of EYL courses at various levels, from EYL 1 to EYL 3, deteriorate. Certain pre-service teachers negatively affect the learning process, particularly in EYL 2 and 3 courses. As a result, it should be a priority for University and EYL lecturers to provide appropriate material. Appropriate materials are those that are not only user-friendly for students but also teachers. It means that content can be delivered to students quickly, and the teacher can easily master the material, ensuring that learning objectives are met (Yüksel and Kavanoz, 2015). It could also be an attempt to help pre-service teachers avoid becoming discouraged during the learning process.

After evaluating why students chose EYL elective courses and their perspectives on the material at each level of EYL courses, the researchers attempted to analyze their beliefs and attitudes toward the EYL teacher profession. In EYL 1, several respondents expressed an interest in becoming EYL teachers. The responses were consistent following their successful completion of the EYL 2 and EYL 3 courses required to become EYL teachers. While some pre-service teachers expressed reservations about becoming EYL teachers during the EYL 1 course, they were sure to become teachers following EYL teaching practices for primary students during the EYL 3 course. On the other hand, some pre-service teachers desired a career in education and then developed doubts, and vice versa. The following section delves into students’ beliefs of the EYL teacher profession.

### Students’ Belief in the English for Young Learners Teacher Profession

The following are the respondents’ beliefs about whether they want to become EYL teachers after completing the courses. Some respondents showed the same belief (consistent) from EYL 1 to EYL 3 courses, who wished to become EYL teachers. Conversely, some students showed very different views (inconsistent) from EYL 1 to EYL 3 times. Some respondents wanted to be teachers and then changed their belief not to become teachers. Some pre-service teachers said they did not want to become EYL teachers, and then they wanted to become teachers after completing the EYL 3 course.

In Table 6 it shows the consistency of students’ beliefs about the EYL teacher profession. After completing all EYL courses, as many as seventeen students consistently wanted to be EYL teachers. Pre-service teachers stated various reasons; several pre-service teachers stated that they were passionate about teaching EYL. After gaining experience teaching EYL in primary school, some students were convinced they wanted to become EYL teachers. Some of them desired careers as private English teachers for young learners. They argued that this is an excellent opportunity in Indonesia, where English as a foreign language remains a prestigious skill. Then, one student consistently expresses an unwillingness to pursue a career in EYL education. This student was one of the students who chose the EYL course to avoid other lecturers. He stated that he enjoyed following the lectures as they progressed from EYL 1 to EYL 3. He also stated that he enjoyed the material about young learner characters. Consistency was also elicited from students who expressed reservations about becoming EYL teachers. Three students expressed reluctance or doubt about becoming EYL teachers after completing all levels of the courses. According to their interviews, the three students chose EYL courses for non-academic reasons, including a desire to work with children.

**TABLE 6:** Students’ belief in teacher EYL profession.

Consistence students’ belief	Number of students	Inconsistence students’ belief	Number of students
W to W	17	DW to W	1
DW to DW	1	W to DW	1
D to D	3	D to W	1
-	-	D to DW	2
-	-	W to D	2
Total	21	Total	7

*To make the data easy to convey, the researchers make several acronyms for several terms such as; W for Wants, D for Doubtful, and DW for Does not Want.*

Additionally, inconsistencies were discovered from several students, including one who initially did not wish to be a teacher. He was, however, very interested in becoming an EYL teacher after completing the EYL 3 course. After analyzing the interview data, he determined that he had a non-academic motivation and wished to avoid other elective courses. Everything changes with time; he enjoys learning EYL theory and concepts, developing lesson plans and media, and he was even more delighted when he was required to practice teaching English to primary school students.

In contrast, one respondent who desired to become a teacher changed her mind after enrolling in the EYL 3 courses. This student was depressed and struggled to adjust to the children. She stated that teaching EYL was more complicated than the theory she had previously learned. Additionally, it is known that one student was inconsistent in his initial goal for not becoming an EYL teacher and then changing his belief for becoming an EYL teacher. He also enjoyed the process of taking EYL classes. Two students also were known to change their beliefs. Initially, they were hesitant to pursue a career as an EYL teacher and eventually decided not to pursue a career as an EYL teacher. They initially chose EYL courses for non-academic reasons. Additionally, the researchers discovered that two students who initially ecstatic developed reservations about pursuing a career as an EYL teacher. The interview data revealed that they were highly interested in learning about EYL teaching concepts. Unfortunately, after practicing teaching elementary school children, they discovered that it was not as simple as learning the theory; they discovered those teaching children were quite exhausting.

This research indicates that university students’ cognition (e.g., belief and perception) changed significantly after completing EYL courses. Among all students who chose EYL, the majority were consistent in their desire to become EYL teachers, while others appeared inconsistent and expressed a desire not to become EYL teachers for various reasons. Cognitive changes observed in previous studies were also both increasing and decreasing. Peacock (2001) discovered changes in trainees’ beliefs about second language learning during their second and third years of study in his research ‘investigating changes in the beliefs about second language learning of trainee ESL teachers’ over a three-year program. According to the findings of these studies, it is theoretically possible that cognition or belief can change due to a variety of factors, including personal experience, professional education, and experience in teaching practice (Borg, 2003, 2015, 2019). The research indicates there has to be an improvement in how the material is presented in all EYL courses. Along with the need for classroom reconstruction to further motivate students to move from theory to practice, students should also be given ample opportunity to choose media and English teaching styles for children during teaching practices. It is consistent with what researchers stated in their research, that students should be provided with a more open environment and that teachers should be supported in their instructional practices (Yuan and Lee, 2014; Chuang et al., 2020; Kim et al., 2020).

## Discussion

For some Indonesians, proficiency in English is considered essential. Therefore, they began learning English as a target language at a young age. Unfortunately, English is not included in the formal education curriculum at the kindergarten and primary school levels. The curriculum in Indonesia has changed, and changes are generally beneficial for improving the quality of education. The curriculum is inextricably linked to the subjects taught at each level of the educational system, including elementary school English. Since English was added to the realm of local content in 1994, almost all schools, both public and private elementary schools, as well as kindergartens, have competed to implement or apply English, resulting in the rapid growth of the language (Sikki et al., 2013; Zein, 2016; Sulistiyo et al., 2020).

Along with English development in elementary schools, learning English encounters difficulties, ensuing in unsatisfactory results, even though each level of education has used the method. It indicates that issues are preventing English from reaching its full potential. One of the issues, namely, the implementation of English, generates controversy among English teachers, particularly those who are excessively demanding of students, causing students to focus exclusively on one subject (English) rather than on other mandatory subjects. Alternatively, learning English is inefficient due to the low quality of English teachers who are also English users as a foreign language. However, English education for EFL children cannot be abandoned due to the increasing demands and the resulting need for English. Maili (2018) discovered through direct interviews with English teachers in elementary schools that they have a reason for the need for English to be implemented so that students can easily transition to high school and are not surprised when accepting English subjects.

For this reason, universities in Indonesia, especially in the English education department, provide Teaching English for Young Learners (TEYL) courses in their curriculum. The purpose of the inclusion of this course is to equip prospective EFL pre-service teachers to teach English to young learners. In one of the well-known private universities in Sidoarjo, East Java includes TEYL courses as a major for final year students, given in stages over three consecutive semesters, EYL 1, EYL 2, and EYL 3. Students certainly have different perceptions and beliefs, from attending the EYL 1 course to completing the EYL 3 course. In this study, the researcher analyzed the differences in their motivation, beliefs, and perceptions during the EYL course learning process. Knowledge, belief, and perception, called cognition, is unobservable factor of someone that cannot be seen (Levine et al., 1993; Borg, 2003). One of the dimensions of cognition is a person’s perceptions and beliefs about something obtained from thinking about something. The process is acquiring knowledge through remembering, analyzing, understanding, judging, and practicing.

This study indicates a shift in the cognition of pre-service teachers from negative to positive and vice versa regarding their belief in becoming English teachers for young learners. Students who initially took this course intending to become EYL teachers changed their minds after three semesters of learning. The findings of this study reinforce Hsu’s (2019) research that student motivation can change during and after the learning process which can affect their cognition. According to Hsu, various intrinsic and extrinsic factors have an effect. For example, intrinsic factors such as student anxiety, hope, self-efficacy, and extrinsic factors such as the environment and the people around them, including the teacher. According to Hsu, gender also affects cognition, with girls generally showing higher motivation levels than boys. On the other hand, in this study, gender differences did not influence changes in students’ cognition. Hsu’s findings about gender are also found in this study, where gender also does not affect the cognition of pre-service teachers. While Hsu focused his research on changes in motivation that affect students’ cognition of learning English as a foreign language, the cognitive changes in this study were on pre-service English teachers for young learners before, during, and after their teacher education.

The potential for students with varying motivations to take this course does not guarantee that they will always have a favorable opinion of TEYL. The pre-service teachers have had different perspectives and beliefs for three consecutive semesters in EYL learning process. Some generate positive perceptions and beliefs from each course, while others generate negative ones. Those with good attitudes are more likely to be interested in teaching English as a foreign language to young learners. Even if they encounter difficulties during the learning process, they are ready to overcome them. They even use their difficulties to train as professional EFL children’s English teachers. The findings of this study simultaneously answer the results of Hollingsworth’s (1989) research, which found that in a longitudinal study of pre-service, teachers tended to always change positively in their beliefs. According to the findings of this study, not all pre-service teachers hold consistent beliefs. Some of them have even been inconsistent with their initial motivation to enroll in the EYL course and their perceptions and beliefs after completing all levels of the EYL courses. The findings of this study became increasingly complex over three semesters when it was found that students who were highly motivated at the beginning of their college developed the low motivation to become English teachers. Interview data revealed that when dealing directly with elementary school students during classroom teaching practice sessions, these pre-service teachers often gave up. Slightly different from Hollingsworth (1989), whose cognition changes for pre-service teachers are only positive. The findings of this study are similar to the results of Yüksel and Başaran (2019) research, which proves that as the learning process progresses, student cognition changes are diverse and dynamic due to various factors that occur from pre-service teachers themselves and also the factors around them during the educational process they undergo.

The motivation and cognition of pre-service teachers varied, which influenced their decision to become English teachers for young learners. According to the findings of this study, not all pre-service teachers hold consistent beliefs. Some of them have even been observed to be inconsistent with their initial motivation for enrolling in EYL courses and their perceptions and beliefs following completion of all EYL method levels. The findings of this study became increasingly complex over the course of three semesters when it was discovered that students who had high motivation at the start of their lectures developed low motivation to become English teachers. The interview data revealed that when dealing directly with elementary school students during classroom teaching practice sessions, students or pre-service teachers of this type frequently gave up.

On the other hand, some pre-service teachers who lacked enthusiasm for TEYL have been known to struggle and complete this tiered course. When confirmed during the interview session, they stated that TEYL initially surprised them but drew them deeper into their role as teachers due to the difficulties inherent in dealing with elementary school students. The findings contradict Stolk and Harari’s (2014) research, which asserts that student motivation can be used to predict active cognitive involvement. The process can alter students’ knowledge, perceptions, and beliefs about a subject. Alieto (2019) emphasized that understanding cognition is a predictor of understanding students’ learning desires. Alito’s statement is consistent with the findings of this study, which indicate that pre-service teachers’ cognitive processes influence their desire to work as English teachers for young learners. By understanding the changes in cognition of pre-service English teaching teachers for young learners in this study, university administrators and teacher educators can develop strategies or steps toward curriculum improvement, particularly in teaching English to young learners in higher education. The findings of this study imply that preparing pre-service teachers to teach English to young learners requires ingenuity for pre-service teachers who are currently learning to absorb the material optimally. Additionally, pre-service English teachers can increase their motivation to learn, thereby achieving the course’s objective of developing English teachers for EFL young learners.

## Conclusion

Overall, this research indicates that English as a foreign language should still be taught to young EFL learners. It accepts qualified candidates for EYL teacher positions in their fields. Numerous English education majors in Indonesia offer EYL courses to their students in the Education Faculty (pre-service teachers). In this study, the students were viewed as persistent throughout the lecture process to become EYL teachers. They are also required to design lesson plans and learning media and provide theories and concepts about EYL. They include teaching practice as a requirement for them to become EYL teachers in the learning desires demonstrate that not all students’ cognition such as belief and perception are consistent. Something has shifted between the rise and fall in their desire to become EYL teachers. These changes are visible from their perspective on all levels of EYL courses. The students whose cognition has shifted positively due to their motivations for teaching EYL and their future aspirations to become EYL teachers. In other words, the amount of time they spend studying EYL does not guarantee they have a strong desire to teach EYL.

Finally, the findings of this study contribute significantly to the understanding that the EYL course is still required in Indonesia’s English department curriculum. The authors acknowledge that their findings may not represent pre-service teachers’ cognition in other universities in non-home English-speaking countries. As a result, additional researchers are expected to conduct similar studies with respondents from various universities. Additionally, this research recommends additional research to analyze the EYL course curriculum’s aspect, whether through the lens of pre-service teachers’ aspirations to become teachers or through other factors that influence students’ perspectives during the lesson.

## Data Availability Statement

The original contributions presented in the study are included in the article/supplementary material, further inquiries can be directed to the corresponding author.

## Author Contributions

YA and SS contributed to the publication process by contributing to the idea, concept, and research design. SA assessed the data’s correctness and verified all writing systems before publication. The version submitted has been read and approved by all authors.

## Conflict of Interest

The authors declare that the research was conducted in the absence of any commercial or financial relationships that could be construed as a potential conflict of interest.

## Publisher’s Note

All claims expressed in this article are solely those of the authors and do not necessarily represent those of their affiliated organizations, or those of the publisher, the editors and the reviewers. Any product that may be evaluated in this article, or claim that may be made by its manufacturer, is not guaranteed or endorsed by the publisher.
